# From Isolation to Application: Designing a Multi-Target Phage Cocktail for Bivalve Safety

**DOI:** 10.3390/microorganisms13122708

**Published:** 2025-11-27

**Authors:** Pedro Costa, Carla Pereira, Jesús L. Romalde, Adelaide Almeida

**Affiliations:** 1 CESAM, Department of Biology, University of Aveiro, Campus Universitário de Santiago, 3810-193 Aveiro, Portugal; csgp@ua.pt; 2 CRETUS, Department of Microbiology and Parasitology, CIBUS—Faculty of Biology, University of Santiago de Compostela, 15782 Santiago de Compostela, Spain; jesus.romalde@usc.es

**Keywords:** bacterial viability, cocktail formulation, phage cocktail, phage isolation, screening assays

## Abstract

Narrow host specificity and bacterial resistance often limit single-phage treatments. Phage cocktails address these challenges by expanding the host range, reducing resistance, and enhancing bacterial inactivation. This study aimed to develop an optimised phage cocktail targeting *Escherichia coli*, *Salmonella enterica* serovar Typhimurium, *Salmonella enterica* serovar Enteritidis, and *Aeromonas hydrophila*, key pathogens in bivalve consumption. Twelve phages were isolated, purified, and screened for bacterial inactivation using resazurin-based viability assays. Host range analysis showed that all phages infected at least one additional bacterial species, with four (phEc4, phSE1, phAh2, phAh4) targeting three of the four bacteria. Cocktail formulation aimed to maximise bacterial reduction while balancing host range expansion with factors such as the risks of resistance development and inter-phage competition. Among the tested combinations, the most effective cocktail consisted of *E. coli* phage phEc3, *S.* Typhimurium phage phST1, *S.* Enteritidis phage phSE1, and *A. hydrophila* phage phAh2. Future studies should evaluate the cocktail’s efficacy *in vitro* and assess both safety and performance *in vivo* in bivalve depuration systems.

## 1. Introduction

Bacteriophages (phages) are viruses that specifically infect bacteria [1] and offer a promising alternative to conventional antibiotics by selectively targeting harmful bacteria while preserving native microbiota [2,3,4,5]. Since their discovery over a century ago, phages have been explored as antibacterial agents in both therapeutic and environmental contexts, with renewed interest driven by the global rise in antimicrobial resistance [6,7,8].

In food safety, phage-based biocontrol has been explored to mitigate contamination risks in various products [9], including bivalve shellfish, which pose significant public health risks due to pathogens such as *Escherichia coli*, *Salmonella enterica* (serovars Typhimurium and Enteritidis), and *Aeromonas hydrophila* [10]. Industrial depuration is widely used to reduce microbial loads in live shellfish before consumption, but its effectiveness against bacterial pathogens varies [10]. Integrating phage biocontrol into depuration could improve bacterial removal efficiency while maintaining sustainability and consumer safety [10]. However, practical implementation faces challenges such as phages’ narrow host range [11,12,13] and the emergence of phage-resistant bacteria [14], both of which limit the success of single-phage applications [1,12,15,16,17,18]. To address these limitations, phage cocktails—combinations of multiple phages—are often employed to expand host range and suppress resistance [19]. These benefits are often described as enhancing a cocktail’s breadth (host range) or depth (resistance suppression) of activity [19].

Several studies have called for cocktails targeting different genera and serovars of bacteria transmitted via bivalves, such as *S.* Enteritidis and *S.* Typhimurium [20,21]. However, the careful selection of appropriate phages is key to success [22]. Host range and bacterial inactivation efficiency are essential criteria during the early stages of phage cocktail development [10]. This is particularly important given that the efficacy of phage cocktails can vary considerably: while some studies show synergistic or additive effects, others report reduced activity—often due to phage competition for the same bacterial receptors [22]. Thus, formulating an effective phage cocktail requires selecting potent candidates and carefully considering their potential interactions [22].

Bacteria employ various resistance mechanisms against phages, including blocking adsorption, degrading or preventing phage nucleic acid entry through restriction-modification systems and the CRISPR/Cas system, and using abortive infection systems to inhibit closely related phages, thereby limiting competitor phage propagation [22,23]. These defences may limit phage propagation and reduce overall cocktail effectiveness [22]. Combining phages that rely on the same receptor can result in antagonistic effects, as a single mutation may render multiple phages ineffective [24].

In contrast, some studies suggest that phage cocktails achieve greater bacterial inactivation than single-phage treatments [25,26], whether targeting the same bacterium [25,27], different strains of a species [28] or multiple bacterial species [26]. Thus, optimising a cocktail requires balancing bacterial reduction, host range, and stability—key challenges in developing effective phage biocontrol strategies.

Several strategies have been developed to counteract bacterial resistance to phages, including phage training, genetic engineering, and cocktails [29,30,31]. In a well-balanced mixture, bacteria resistant to one phage may remain susceptible to others [32], enhancing reliability and reducing treatment failure [33], a concept that could also extend to cocktails targeting multiple bacterial species [26]. However, randomly assembled cocktails can be inconsistent due to negative phage interactions [11].

An alternative strategy is to use fewer phages if individual phages can infect multiple bacterial species [34,35]—commonly referred to as polyvalence [36,37]. However, the chances of isolating effective polyvalent phages decrease with broader bacterial diversity, especially across Gram classifications [19]. For this reason, cocktails combining phages that target distinct receptors are preferred [38], though identifying these phages requires significant time, labour, and screening [24].

Since phages recognise their bacterial hosts through specific surface receptors, cocktail design often requires a degree of personalisation [13]. Given these complexities, several studies advocate for an empirical approach—testing diverse phage combinations with overlapping host ranges to identify the most effective mixes without the need for full receptor characterization [22].

Conventional methods for assessing phage–bacteria dynamics, such as colony counting and optical density (OD) measurements, are either too time-consuming or lack precision for large-scale screening [39]. To overcome these limitations, we employed the resazurin cell viability assay—a high-throughput method that simultaneously evaluates multiple bacteria–phage interactions [39]. This assay relies on the reduction of blue, non-fluorescent resazurin to the pink, fluorescent compound resorufin by metabolically active cells, allowing bacterial viability to be quantified by fluorescence or absorbance as an indirect measure of bacterial inactivation [39]. This approach enabled us to identify cocktails with broader host ranges, designed to delay resistance and maintain or improve upon single-phage efficacy.

In this study, we isolated phages targeting *E. coli*, *S. enterica* serovars Typhimurium and Enteritidis, and *A. hydrophila*. These were formulated into cocktails, and their antibacterial activity was evaluated *in vitro* using a viability-based approach. Our aim was to select reliable candidates for further *in vitro* and *in vivo* application in bivalve depuration systems.

## 2. Materials and Methods

### 2.1. Bacterial Strains and Growth Conditions

The bacterial strains used in this study as phage hosts included *E. coli* (ATCC 25922), *S.* Typhimurium (ATCC 13311), *S.* Enteritidis (CVB) and *A. hydrophila* (ATCC 7966). *A. hydrophila* (ATCC 7966), *E. coli* (ATCC 25922) and *S*. Typhimurium (ATCC 13311) were purchased from the ATCC collection. *S*. Enteritidis (CVB) is a food sample isolate generously provided by Controlvet Laboratory.

Fresh bacterial cultures were preserved on Tryptic Soy Agar (TSA; Liofilchem, Roseto degli Abruzzi, Italy) at 4 °C. Before each assay, an isolated colony was aseptically transferred to Tryptic Soy Broth (TSB; Liofilchem, Roseto degli Abruzzi, Italy) and incubated overnight at 25 °C. One hundred microliters of this culture were aseptically transferred to fresh TSB and incubated overnight at 25 °C until an optical density (OD_600_ of 0.8), corresponding to approximately 10^9^ cells per mL, was reached.

### 2.2. Phage Isolation, Host Range and Purification

All phages were isolated from the Aveiro sewage network (station EEIS9 of the Multi-Sanitation System of Ria de Aveiro—SIMRIA). Sewage water samples were filtered through 0.45 µm pore size polycarbonate membranes (Millipore, Bedford, MA, USA). The resulting filtrate was added to a double-strength TSB medium, along with 1 mL of fresh cultures of each target host bacterium: *E. coli* (ATCC 25922), *S*. Typhimurium (ATCC 13311), *S*. Enteritidis (CVB), and *A. hydrophila* (ATCC 7966). These mixtures were incubated at 25 °C with shaking at 80 rpm for 18 h to promote phage–bacteria interactions, thereby accelerating the lytic cycle and enhancing phage amplification. Following incubation, samples were filtered through 0.2 µm membranes to remove bacterial cells. Phage presence was assessed using the double-layer agar method [40]. Briefly, 100 µL of phage lysate was mixed with 300 µL of an overnight bacterial culture (~10^8^ colony-forming units per millilitre [CFU/mL]) and added to 5 mL of molten soft agar (0.6% *w*/*v*, pre-warmed to 50 °C), which was then overlaid onto TSA plates (1.5% *w*/*v*). Plates were incubated at 25 °C and examined for lytic plaques after 12 h.

Each unpurified phage stock was tested against the other target bacteria to identify phages capable of infecting multiple bacterial hosts. Phages with overlapping host infections were identified, and 22 phages were isolated. Individual plaques were picked and transferred to a TSB medium containing fresh cultures of the corresponding host bacteria. These mixtures were incubated at 25 °C with shaking at 80 rpm for 18 h, as previously described, to allow phage propagation. After incubation, the cultures were centrifuged at 10,000× *g* for 10 min at room temperature to pellet bacterial cells, and the resulting supernatant was used for subsequent rounds of phage isolation. Three successive single-plaque isolation cycles were performed to obtain pure phage stocks. Lysates were centrifuged at 10,000× *g* for 10 min at 4 °C to remove bacterial debris, and the purified phage stocks were stored at 4 °C. Of the 22 isolated phages, 12 were successfully purified. Phage titres were determined using the double-layer agar method [40], as previously described. Plates were incubated at 25 °C for 8 h, and the number of lytic plaques was counted. The results were expressed as plaque-forming units per millilitre (PFU/mL). Phages phEc1, phEc2, phEc3, phST1, phST2, and phST3 were previously described in a separate study [39]; however, all phages reported here, including these, were originally isolated using the protocol detailed above.

### 2.3. Bacterial Killing Curves

Bacterial inactivation was assessed using *E. coli* (ATCC 25922), *S.* Typhimurium (ATCC 13311), *S.* Enteritidis (CVB), and *A. hydrophila* (ATCC 7966) in TSB at 25 °C, using a multiplicity of infection (MOI) of 10, based on each phage’s original host bacterium, to achieve ≥99.99% infection efficiency in phage–bacteria systems [41]. Each assay was initiated with approximately 1 × 10^7^ CFU/mL of bacteria and 1 × 10^8^ PFU/mL of phages. For phage cocktails, each phage was added at an equal concentration relative to its primary host, ensuring consistent MOI and minimizing potential dominance or suppression effects. Only two-phage cocktail combinations were tested in this study. While the twelve isolated phages allowed for possible three- and four-phage formulations, testing all permutations would have required a prohibitively large and complex matrix of experimental conditions. This complexity, combined with the need for replication and continuous viability monitoring over 24 h, led us to focus on two-phage combinations as a tractable and informative first step. A bacterial control (no phage added) was included for each assay, and all samples—control and test—were incubated under identical conditions.

Bacterial viability was assessed at each timepoint (0, 2, 4, 6, 8, 10, and 24 h) using a resazurin-based fluorescence assay, as previously described [39]. At each timepoint, 100 µL of each test or control sample was transferred into black, opaque 96-well plates, followed by the addition of 20 µL of resazurin solution (0.15 mg/mL; Fluorochem Ltd., Glossop, UK). Blank wells containing only sterile media and resazurin were included to correct for background fluorescence; their relative fluorescent unit (RFU) values were subtracted from the sample readings.

To facilitate resazurin reduction by metabolically active bacteria, plates were incubated at 37 °C in the dark for 2 h without shaking. At the 24 h timepoint, only a 10-min incubation was required due to elevated metabolic activity. Although the main phage inactivation assays were conducted at 25 °C, this brief temperature shift during the assay is unlikely to have influenced bacterial growth [42,43,44]. Furthermore, since all samples—including controls—underwent the same treatment, comparative viability assessments remain valid. All experiments were performed in triplicate.

### 2.4. Statistical Analysis

Statistical analysis was conducted using GraphPad Prism software 9 (San Diego, CA, USA). The normality of the data was assessed with the Kolmogorov–Smirnov test, while Levene’s test was employed to confirm the homogeneity of variance. Two-way ANOVA with repeated measures, followed by Tukey’s multiple comparison post-hoc test, was used to evaluate significant differences between RFU values (see Section 3.2). A *p*-value of less than 0.05 was considered statistically significant.

## 3. Results

### 3.1. Phage Isolation and Purification

Phages phEc1, phEc2, phEc3 [39], and phEc4 were isolated using *E. coli* as host; phages phST1, phST2, and phST3 were isolated using *S.* Typhimurium as host [39]; phages phSE1, phSE2, and phSE3 were isolated using *S.* Enteritidis as host; and phages phAh2 and phAh4 were isolated using *A. hydrophila* as host. High titre suspensions (>10^9^ PFU/mL) were achieved for all phages. Host range revealed that *E. coli*, *S.* Typhimurium, *S*. Enteritidis and *A. hydrophila* displayed lysis plates on 11, 11, 4 and 2 of the 12 phages tested, respectively (Table 1).

### 3.2. Phage Screening Assays

Since phages can infect and inactivate bacteria other than their original hosts [26], screening assays were performed to further evaluate the effectiveness of individual phage suspensions and various phage combinations in bacterial inactivation. As such, we employed the resazurin cell viability assay [39] to assess the inactivation profiles of each phage and different phage combinations to identify the most effective phages for inclusion in the previously delineated optimal cocktail.

#### 3.2.1. *E. coli* Inactivation Screening

**Figure 1:**
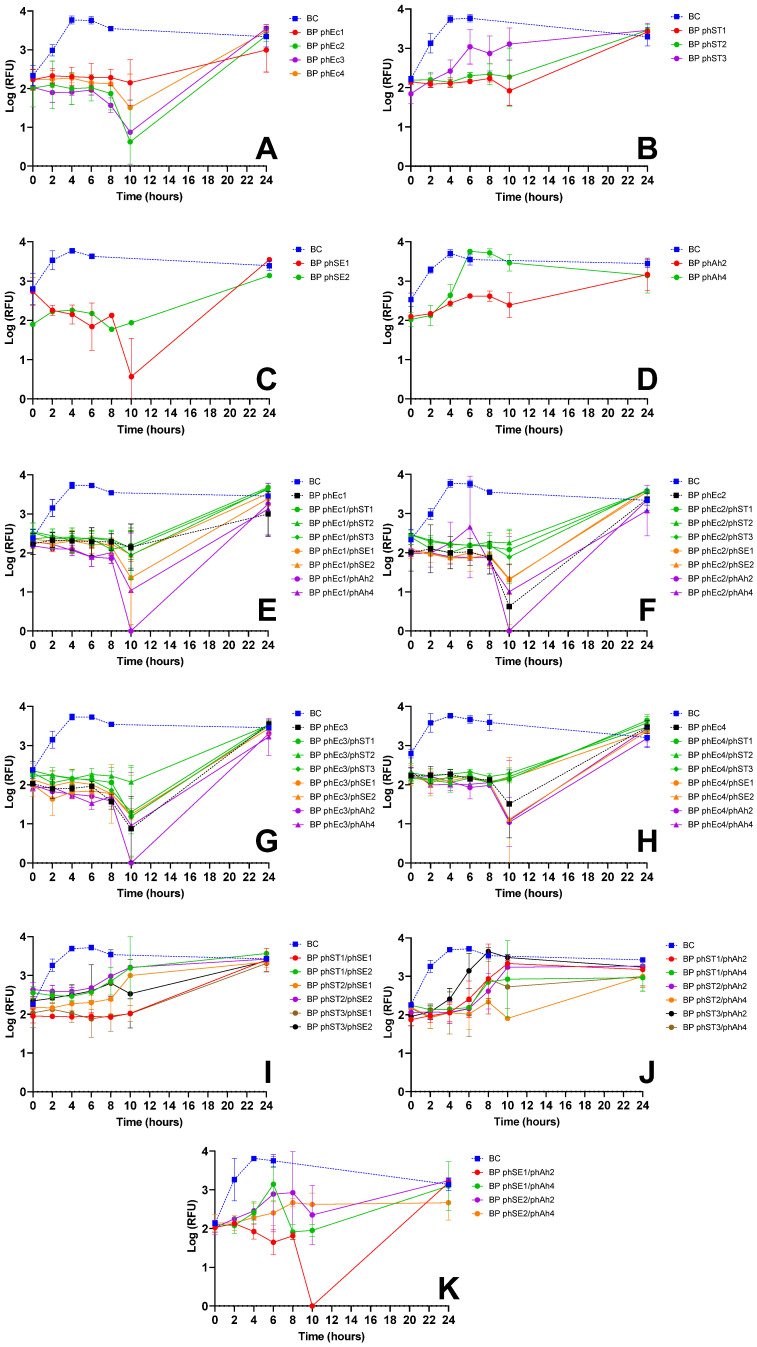
Inactivation of *E. coli* over 24 h using single-phage suspensions and two-phage cocktails at an MOI of 10, evaluated via bacterial viability through resazurin-based cell viability assay. Panels show the effect of (**A**) *E. coli* phages (phEc1, phEc2, phEc3, phEc4); (**B**) *S*. Typhimurium phages (phST1, phST2, phST3); (**C**) *S*. Enteritidis phages (phSE1, phSE2, phSE3); and (**D**) *A. hydrophila* phages (phAh2, phAh4); *E. coli* phages (black) and cocktails combining each *E. coli* phage, phEc1 (**E**), phEc2 (**F**), phEc3 (**G**), and phEc4 (**H**) with other *S.* Typhimurium (green), *S*. Enteritidis (orange) and *A. hydrophila* (purple) phages; (**I**) cocktails combining *S*. Typhimurium phages with *S.* Enteritidis phages; (**J**) cocktails combining *S*. Typhimurium phages with *A. hydrophila* phages; and (**K**) cocktails combining *S*. Enteritidis phages with *A. hydrophila* phages. RFU: relative fluorescence unit, an indicator of metabolic activity. BC: bacteria control; BP: bacteria plus phage. Values represent the mean of three independent assays; error bars represent the standard deviation.

Screening assays were performed with all *E. coli*-infecting phages and cocktails combining two phages from different bacterial hosts (Figure 1, Table A1). Single-phage suspensions reduced *E. coli* viability for 6 to 24 h (Figure 1A–D), with phages phEc2, phEc3 (Figure 1A), phST1, phST2 (Figure 1B), phSE1 (Figure 1C), and phAh2 (Figure 1D) demonstrating the strongest inactivation, reducing bacterial viability by approximately 1 to 2.5 log RFU relative to the control (ANOVA, *p* < 0.05).

Cocktails combining *E. coli* phages with *S.* Typhimurium (Figure 1E–H, green) or *S.* Enteritidis phages (Figure 1E–H, orange) generally showed no improvement compared with each *E. coli* phage alone (Figure 1E–G, black), except for the cocktail phEc1/phSE1 (Figure 1E), which enhanced inactivation by ~1 log RFU (ANOVA, *p* < 0.05). In contrast, combinations such as phEc3/phST2 (Figure 1G), phEc2 with *S.* Typhimurium phages (Figure 1F, green) and phEc4 with *S.* Typhimurium phages (Figure 1H, green) showed reduced efficacy. Adding *A. hydrophila* phages enhanced inactivation by ~1 to 2 log RFU (ANOVA, *p* < 0.05), particularly phages phAh2 (Figure 1E–G) and phAh4 (Figure 1E), or maintained similar profiles (Figure 1H, purple).

Cocktails without *E. coli* phages, including combinations of *S.* Typhimurium, *S.* Enteritidis, and *A. hydrophila* phages, frequently showed increased bacterial viability after 10 h (Figure 1I,J). However, combinations such as phST1/phSE1 (Figure 1I), phST2/phAh4 (Figure 1J), and phSE1/phAh2 (Figure 1K) exhibited 1.5–3 log RFU reductions compared to controls (ANOVA, *p* < 0.05).

#### 3.2.2. *S.* Typhimurium Inactivation Screening

**Figure 2:**
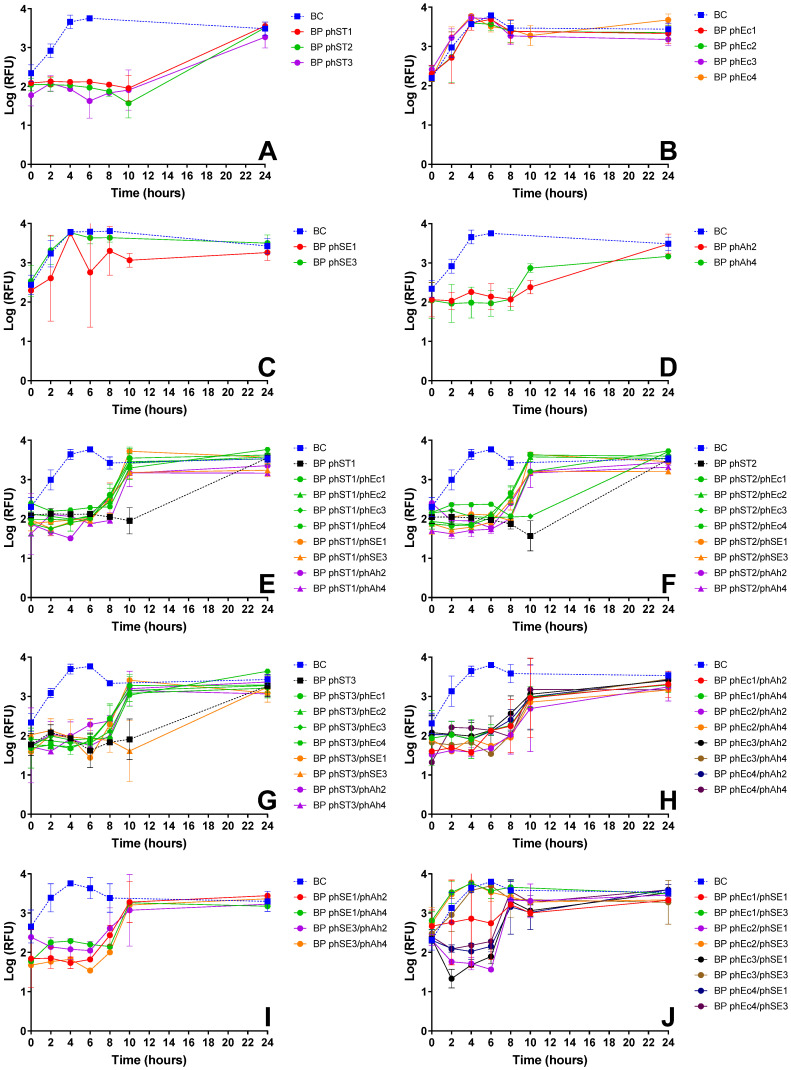
Inactivation of *S*. Typhimurium over 24 h using single-phage suspensions and two-phage cocktails at an MOI of 10, evaluated via bacterial viability through resazurin-based cell viability assay. Panels show the effect of (**A**) *S*. Typhimurium phages (phST1, phST2, phST3); (**B**) *E. coli* phages (phEc1, phEc2, phEc3, phEc4); (**C**) *S*. Enteritidis phages (phSE1, phSE2, phSE3); and (**D**) *A. hydrophila* phages (phAh2, phAh4); *S.* Typhimurium phages (black) and cocktails combining each *S.* Typhimurium phage, phST1 (**E**), phST2 (**F**), and phST3 (**G**), with other *E. coli* (green), *S*. Enteritidis (orange) and *A. hydrophila* (purple) phages; (**H**) cocktails combining *E. coli* phages with *A. hydrophila* phages; (**I**) cocktails combining *S*. Enteritidis phages with *A. hydrophila* phages; and (**J**) cocktails combining *E. coli* phages with *S*. Enteritidis phages. RFU: relative fluorescence unit, an indicator of metabolic activity. BC: bacteria control; BP: bacteria plus phage. Values represent the mean of three independent assays; error bars represent the standard deviation.

Screening assays were performed with all *S.* Typhimurium-infecting phages and cocktails combining two phages from different bacterial hosts (Figure 2, Table A2). Single-phage suspensions of *S.* Typhimurium and *A. hydrophila* phages produced ~1.5 log RFU reductions over 24 h (Figure 2A,D, ANOVA, *p* < 0.05), while *E. coli* and *S.* Enteritidis phages showed no effect (Figure 2B,C).

Cocktails combining *S.* Typhimurium phages with phages targeting other bacteria generally showed an earlier rebound in bacterial viability after 10 h, compared to single-phage treatments (Figure 2E–G). However, certain combinations such as phST2/phEc3 (Figure 2F) and phST3/phSE3 (Figure 2G) maintained inactivation comparable to that of *S.* Typhimurium phages alone (~1.5 log RFU, ANOVA, *p* < 0.05), indicating no loss of efficacy.

For cocktails without *S.* Typhimurium phages, combinations of *A. hydrophila* phages with *E. coli* or *S.* Enteritidis phages produced similar inactivation profiles with increased bacterial viability after 10 h (Figure 2H,I). The combination phEc3/phSE1 exhibited higher early inactivation (~2 log RFU, ANOVA, *p* < 0.05), whereas other *E. coli*-*S.* Enteritidis phage cocktails, such as phEc1/phSE3, phEc2/phSE3, and phEc3/phSE3, showed no additional effect on bacterial viability (Figure 2J).

#### 3.2.3. *S.* Enteritidis Inactivation Screening

**Figure 3:**
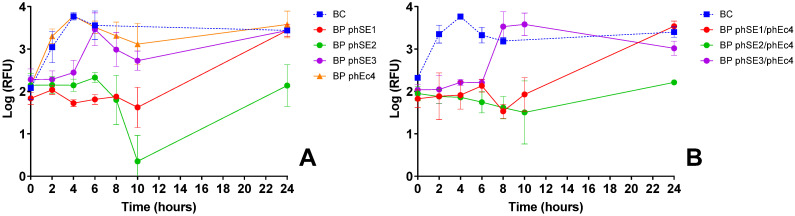
Inactivation of *S*. Enteritidis over 24 h using single-phage suspensions and two-phage cocktails at an MOI of 10, evaluated via bacterial viability through resazurin-based cell viability assay. Panels show the effect of: (**A**) *S*. Enteritidis phages (phSE1, phSE2, phSE3) and *E. coli* phage phEc4; and (**B**) cocktails combining each *S*. Enteritidis phage (phSE1, phSE2, phSE3) with the *E. coli* phage phEc4. RFU: relative fluorescence unit, an indicator of metabolic activity. BC: bacteria control; BP: bacteria plus phage. Values represent the mean of three independent assays; error bars represent the standard deviation.

Screening assays were performed with all *S.* Enteritidis-infecting phages and cocktails combining both (Figure 3, Table A3). Single-phage suspensions of *S.* Enteritidis phages effectively reduced bacterial viability by ~1.5–2 log RFU (ANOVA, *p* < 0.05), except phSE3, which showed minimal effect, with RFU values similar to the control after 6 h (Figure 3A). While phEc4 could infect *S.* Enteritidis, it did not impact bacterial viability (Figure 3A).

In combination assays, phEc4 either extended the effectiveness of *S.* Enteritidis phages, as seen with phSE3/phEc4 after 6 h, or reduced their effectiveness by ~1 log RFU, as observed with phSE2/phEc4 after 10 h (Figure 3B, ANOVA, *p* < 0.05).

## 4. Discussion

Phage cocktails are a promising approach to overcoming the limitations of single-phage treatments [11], particularly regarding narrow host specificity [11,12,13] and bacterial resistance [14]. Additionally, they can achieve greater bacterial inactivation than single-phage treatments [25,26]. However, their success depends on selecting complementary phages [38]. This study aimed to develop an optimised phage cocktail against *E. coli*, *S*. Typhimurium, *S*. Enteritidis, and *A. hydrophila*—key pathogens in bivalve consumption. Using resazurin-based viability assays, we identified a cocktail that matched or exceeded single-phage performance, targeted multiple bacteria, and minimised resistance development.

To identify phages capable of infecting more than one target bacterium, we used an isolation workflow that allowed us to recover candidates exhibiting activity beyond their original host (Section 2.2). Host range analysis (Table 1) showed that all phages infected at least one additional bacterial species, with some capable of targeting three of the four bacteria, which is expected for closely related enteric bacteria such as *E. coli* and *S.* Typhimurium due to genomic and ecological similarities [45]. This approach provided a practical means to narrow down and identify viable candidates for cocktail formulation [26] and subsequent testing and optimization. Similar strategies to identify broad-host-range phages from environmental samples have been reported [46,47], though with varying methodologies and goals. However, relying on phages with broader host ranges, like phAh2, poses important challenges. Broad-spectrum infectivity does not guarantee uniform efficacy across hosts, as discussed below; phages may exhibit reduced adsorption efficiency or lower burst size on secondary hosts [35]. Furthermore, such phages may exert selective pressure that promotes resistance in unintended bacterial populations [48,49]. Thus, while broad-host-range phages can simplify cocktail design, their inclusion must be supported by robust, context-specific efficacy data—an aspect we explore in the following sections.

To generate these efficacy data, after isolation and purification, each phage was then tested for its ability to inactivate target bacteria. In most cases, phages effectively reduced the viability of non-host bacteria (Figure 1A–D and Figure 2A,D). Similar findings were reported by Costa et al. (2019) [26], where an *E. coli* phage inactivated *S.* Typhimurium, and a *S.* Typhimurium phage inactivated *E. coli*. However, some phages unexpectedly formed plaques in host range assays but failed to reduce bacterial viability in liquid cultures. Notably, *E. coli* and *S.* Enteritidis phages showed no effect on *S.* Typhimurium viability (Figure 2B,C), and phEc4 failed to inactivate *S.* Enteritidis (Figure 3A) despite all forming plaques in host range assays (Table 1). This discrepancy may be attributed to a low efficiency of plating (EOP), as supported by follow-up testing of the selected phages, which showed low or undetectable EOP on infectable but non-host bacteria [50]. In such cases, phage replication and subsequent inactivation may occur at such low levels that any effect is masked by rapid bacterial regrowth at later assay stages [51,52]. To explore this, we conducted a preliminary, CFU-based inactivation assay using phSE3 against *S.* Typhimurium. The initial PFU count was very low, and CFU reduction was only observed at a single time point, with no differences from the untreated control at any other time. Although not conclusive, this supports the hypothesis that low EOP may hinder detectable inactivation in RFU-based assays, especially when bacterial regrowth dominates. While resazurin provides a rapid, high-throughput screening tool, it primarily measures metabolic activity, which may not always correlate directly with viable cell counts [39]. This can result in false positives (e.g., metabolically active but non-replicating cells), false negatives (e.g., low metabolic activity despite viability) [53], or early phage effect at the beginning of the assays on bacterial metabolic activity during the incubation period [39], as seen in some instances (Figure 1C and Figure 2H). Such limitations become especially relevant when phage replication is inefficient, reinforcing the importance of complementary quantification methods. Although CFU enumeration has been applied to the selected phages [50], further work using additional confirmatory assays—such as adsorption rate measurements, one-step growth curves, and time-kill assays—will be necessary to validate findings across the entire phage panel.

Building on these single-phage assessments, we next examined how phages performed in combination. Phage cocktails sometimes provided enhanced bacterial suppression relative to individual phages, but this benefit depended strongly on the compatibility of specific phage partners (Figure 2J and Figure 3). Similar observations have been reported by Costa et al. (2019) [26], who also noted improved bacterial control when certain phages were combined. Such enhanced performance is often attributed to synergistic interactions, in which one phage facilitates or amplifies the activity of another through complementary infection strategies [13,54]. For example, Schmerer et al. (2014) [54] described synergy arising from enzymatic modification of the bacterial surface that increased access for a partner phage, while Regeimbal et al. (2016) [13] showed that selective pressure imposed by one phage can generate variants that become more susceptible to another. Mechanisms of this kind may underline the improved suppression observed in some combinations in this study. However, because synergy is highly context-dependent and can emerge from multiple interacting biological processes, future work will be needed to determine the specific factors contributing to these effects.

Despite some promising results, most phage combinations either produced inactivation profiles similar to the most effective single-phage treatment (Figure 1E–H, Figure 2E–G and Figure 3B), showed reduced efficacy compared to single-phage treatments—as observed for the phEc3/phST2 combination (Figure 1G), highlighting potential inter-phage antagonism within multicomponent mixtures—or led to earlier bacterial regrowth (Figure 1F–J, Figure 2E–I and Figure 3B). The latter was supported by visible turbidity accompanying the late-stage fluorescence rise, confirming biomass recovery rather than a resazurin artifact, which we interpreted as an indirect indicator of resistance development [55]. These findings are consistent with prior reports that randomly assembled cocktails often display variable effectiveness due to interactions among component phages [11,22] and reinforce the importance of an empirical screening workflow, since phage–phage interactions frequently diverged from expectations based solely on host-range profiles or individual efficacy.

One likely explanation is competition between phages targeting the same bacterial surface receptor, which can render cocktails ineffective if bacteria mutate that receptor [24]. Additional resistance mechanisms may also contribute, including prophage-encoded defences, mobile genetic elements, intercellular communication, quorum sensing, and superinfection exclusion [22]. Given the overlapping host ranges of the phages, quorum sensing and superinfection exclusion are particularly relevant. Quorum sensing allows bacteria to modulate susceptibility based on population density, downregulating phage receptors and upregulating defence systems such as CRISPR–Cas in response to phage detection [22]. Superinfection exclusion, a prophage-encoded mechanism, prevents entry of closely related phages once one has already infected the host, inhibiting secondary infections and suppressing competing phages [22]. Although direct phenotypic or genetic confirmation of resistance was beyond the scope of this study—including receptor profiling or assays to monitor resistance emergence—the earlier or more pronounced regrowth observed in some cocktails suggests that inter-phage interactions and these resistance mechanisms likely influenced treatment outcomes. Future work will aim to isolate and characterize resistant strains to better understand these dynamics and assess how bacterial exposure to the phage cocktail affects virulence, growth dynamics, surface receptors, and potential genomic or functional changes in the phages themselves. Nonetheless, mitigation strategies such as sequential phage application, selection of geographically diverse phages, or inclusion of naturally co-evolved “guard” phages may offer promising avenues to minimize resistance [22].

As summarized in Table 2, optimizing a phage cocktail requires balancing bacterial inactivation, host range [19], and resistance management [22] while minimising unnecessary complexity [56]. Several highly effective combinations were identified, but not all were suitable for inclusion in the final formulation. Selection was guided by three main considerations: (1) maximizing bacterial suppression, (2) targeting multiple pathogens, and (3) minimizing phage interactions that could reduce efficacy. For *E. coli*, phEc3 consistently appeared in top-performing cocktails, demonstrating strong inactivation both alone and in combination with other phages. Although phEc4 exhibited a broad host range, it was ultimately excluded because it did not enhance bacterial suppression and, in some cases, reduced inactivation when combined with other phages. For *S*. Typhimurium, several phages performed well, but no single candidate stood out across all conditions. Given its frequent presence in effective *E. coli*-targeting cocktails, phST1 was selected to ensure strong suppression while maintaining synergy with other phages. For *S*. Enteritidis, phSE2 showed slightly higher efficacy in single-phage treatments; however, phSE1 was chosen due to its broader host range, maximizing overall cocktail coverage. For *A. hydrophila*, both phAh2 and phAh4 showed promise. Although phAh4 exhibited slightly better *E. coli* inactivation when combined with *S.* Typhimurium phages, phAh2 was selected for its broader efficacy across target bacteria. Comparative analysis confirmed that phAh2 provided similar inactivation without adding complexity. The final cocktail—comprising phEc3 (*E. coli*), phST1 (*S.* Typhimurium), phSE1 (*S.* Enteritidis), and phAh2 (*A. hydrophila*)—represents a carefully balanced combination of effectiveness, host coverage, and resistance mitigation.

Following selection of the four candidate phages, subsequent work focused on validating their suitability for application. This study represents the preliminary screening phase of a broader project aimed at developing an effective phage cocktail for bivalve depuration. The selected phages then underwent comprehensive morphological, genomic, and physiological characterization, including genome sequencing, annotation, and taxonomic classification [50]. The genomes were screened to confirm the absence of genes associated with lysogeny, virulence, or antimicrobial resistance. The phages—renamed PCEc3, PCST1, PCSE1, and PCAh2—were subsequently tested as a cocktail in targeted *in vitro* assays using CFU enumeration and evaluated under relevant environmental conditions, including seawater stability, pH tolerance, and thermal resilience [57]. Although incomplete lysis was observed under the controlled, nutrient-rich conditions of the *in vitro* assay, this does not necessarily indicate reduced efficacy in practical settings, where bacterial densities, nutrient availability, the presence of non-target microorganisms, and environmental conditions differ markedly [58,59,60]. Indeed, the cocktail’s efficacy and persistence were confirmed in *in vivo* bivalve depuration trials [57], indicating that regrowth observed *in vitro* reflects optimal laboratory conditions and does not compromise its applied potential. Together, these findings lay the groundwork for a robust and practical approach to phage cocktail development for food safety applications.

## 5. Conclusions

This study presents a systematic framework for phage cocktail design that integrates host-range screening, cross-species efficacy testing, and assessment of phage–phage interactions. Our findings show that empirical cocktail screening can reveal both synergistic and antagonistic phage interactions that are not predictable from host-range data alone. These results demonstrate that effective cocktail formulation requires balancing bacterial suppression, breadth of activity, and resistance management through rational trade-offs—maximizing efficacy and coverage while minimizing complexity and antagonism. The proposed workflow provides a transferable template for designing multi-target phage cocktails for food safety and environmental biocontrol applications. Future work should validate these findings under realistic depuration conditions, integrating genomic and environmental stability analyses to confirm safety and performance. Ultimately, this approach advances the practical implementation of phage-based strategies to enhance microbial safety in aquaculture and shellfish production.

## Figures and Tables

**Table 1:** Phage host ranges.

Phages	*E. coli*	*S.* Typhimurium	*S.* Enteritidis	*A. hydrophila*
phEc1	H	●	X	X
phEc2	H	●	X	X
phEc3	H	●	X	X
phEc4	H	●	●	X
phST1	●	H	X	X
phST2	●	H	X	X
phST3	●	H	X	X
phSE1	●	●	H	X
phSE2	●	X	H	X
phSE3	X	●	H	X
phAh2	**●**	●	X	H
phAh4	**●**	●	X	H

H—Isolation host. ●—Lysis plaques. X—No lysis plaques.

**Table 2:** Phage Selection Based on Efficacy vs. Host Range Trade-Offs.

Target Bacterium	Selected Phage	Efficacy Highlights	Host Range Justification	Reason for Selection over Alternatives
*E. coli*	phEc3	Strong inactivation alone (Figure 1A) and in cocktails (e.g., phEc3/phAh2); no antagonism observed	Good specificity without cross-host antagonism	More effective than other *E. coli* phages; synergetic in cocktails
*S*. Typhimurium	phST1	Robust suppression; maintained efficacy in multiple cocktails (e.g., phST1/phSE1); did not induce early regrowth	Functional in both single and combination treatments	Preferred over phST2, which showed antagonism with phEc3 (Figure 1G)
*S*. Enteritidis	phSE1	Effective in single and combo assays (Figure 1C,E,K); e.g., phEc1/phSE1 and phSE1/phAh2 showed enhanced activity	Broader host range than phSE2 or phSE3, aligning with multi-pathogen coverage goal	Better synergy and coverage than more potent but narrower phSE2
*A. hydrophila*	phAh2	Strong enhancement in cocktails with phEc3 and phSE1 (Figure 1E–G); consistent inactivation across targets	Broader efficacy across host bacteria compared to phAh4	Chosen over phAh4 for broader effectiveness with fewer phage–phage antagonisms

## Data Availability

The original contributions presented in this study are included in the article. Further inquiries can be directed to the corresponding authors.

## Abbreviations

The following abbreviations are used in this manuscript:

CFU	Colony-Forming Unit
EOP	Efficiency of Plating
MOI	Multiplicity Of Infection
OD	Optical Density
PFU	Plaque-Forming Unit
RFU	Relative Fluorescent Unit
TSA	Tryptic Soy Agar
TSB	Tryptic Soy Broth

## Appendix A

**Table A1:** Host infectivity * of phages against *E. coli*.

Phages	phEc1	phEc2	phEc3	phEc4	phST1	phST2	phST3	phSE1	phSE2	phAh2	phAh4
phEc1	10	-	-	-	-	-	-	-	-	-	-
phEc2	ND	10	-	-	-	-	-	-	-	-	-
phEc3	ND	ND	10	-	-	-	-	-	-	-	-
phEc4	ND	ND	ND	10	-	-	-	-	-	-	-
phST1	10	10	10	10	10	-	-	-	-	-	-
phST2	10	10	10	10	ND	10	-	-	-	-	-
phST3	10	10	10	10	ND	ND	8	-	-	-	-
phSE1	10	10	10	10	10	8	10	10	-	-	-
phSE2	10	10	10	10	8	8	10	ND	10	-	-
phAh2	10	10	10	10	8	8	6	10	10	10	-
phAh4	10	10	10	10	8	10	8	10	10	ND	4

* Host infectivity is measured in hours of hold time, representing the duration of phage suppression on bacterial viability in liquid culture.  Hold Time (Hours).

**Table A2:** Host infectivity * of phages against *S.* Typhimurium.

Phages	phST1	phST2	phST3	phEc1	phEc2	phEc3	phEc4	phSE1	phSE3	phAh2	phAh4
phST1	10	-	-	-	-	-	-	-	-	-	-
phST2	ND	10	-	-	-	-	-	-	-	-	-
phST3	ND	ND	10	-	-	-	-	-	-	-	-
phEc1	8	8	8	0	-	-	-	-	-	-	-
phEc2	8	8	8	ND	0	-	-	-	-	-	-
phEc3	8	10	8	ND	ND	0	-	-	-	-	-
phEc4	8	8	8	ND	ND	ND	0	-	-	-	-
phSE1	8	8	8	6	6	6	6	2	-	-	-
phSE3	8	8	10	0	6	0	6	ND	0	-	-
phAh2	8	8	8	8	8	8	8	8	8	10	-
phAh4	8	8	8	8	8	8	8	8	8	ND	10

* Host infectivity is measured in hours of hold time, representing the duration of phage suppression on bacterial viability in liquid culture.  Hold Time (Hours).

**Table A3:** Host infectivity * of phages against *S.* Enteritidis.

Phages	phSE1	phSE2	phSE3	phEc4
phSE1	10	-	-	-
phSE2	ND	10	-	-
phSE3	ND	ND	4	-
phEc4	10	10	6	ND

* Host infectivity is measured in hours of hold time, representing the duration of phage suppression on bacterial viability in liquid culture.  Hold Time (Hours).
